# James Vick's Floral Guide for 1887

**Published:** 1887-02

**Authors:** 


James Vick's Floral Guide for 1887, is a beautiful volume which it would be extremely difficult to surpass, as it is gotten up in the highest style of art, and contains much valuable information on every branch of the floral art Flowers, shrubs and vegetables are treated of, as only those conversant with the most improved methods of their cultivation can describe such processes. The seeds, plants, &c., from this long established house, have, for quite a number of years, enjoyed the highest reputation as proving reliable and perfect.

